# Shall We Have One Standard of Morals? What Shall the New Standard Be?

**Published:** 1913-07

**Authors:** 


### Shall We Have One Standard of Morals? What Shall the New Standard Be?

Yes, we are going to have one standard of morals; the passing of the double standard is inevitable. Woman is coming into her own and with the coming she is bringing changes that could not exist under the old regime but that she herself will help to make possible and practical under the new.

The elimination of the double standard is so evident that men admit it is coming, and coming rapidly. The man of the world who sees merely the surface of things declares that woman is not being elevated by this process of elimination, but is being lowered to the standard formerly fixed by man himself. In this he is mistaken Where there is one idle, dissolute woman there are hundreds who go quietly about their business, and the sensational world takes no cognizance of their existence. Everywhere vice is being exposed and women are crying out against the double standard that permits a man to send his children through life blinded, his wife to the operating table or the invalid's chair, while he tries to shift upon God the responsibility and censures a Divine Providence for bringing suffering to his loved ones.

We behold on the social horizon the dawning of a new day, when sin shall know no sex. There is no need to fear for what may overtake us under this new order of things. Woman—and when I say woman I speak of the woman who thinks and works and prays—has never lowered the standard of anything to which she has given her earnest attention. On the contrary, she has always elevated and refined whatever she has turned her hand to do.

The existence of the double standard is the result of the fact that the world today is a man-made world and everything in it bears the imprint of his thoughts, his ideals, his tyranny.

Even woman herself is the creature of his making. Fitted into the molds of his conception, bounded by his narrow limitations for her, is it any wonder that she became a mere parasite? It was his idea that she did not need book learning. Her place was at home with her children. All that was necessary was for her to be a good housekeeper and a good mother. He had brains enough for the family and when he came home at night did not care to encourage any mental discussion on the part of his wife. Did not he come home to rest?

As the maker of social laws he would not take any chance of feeding and clothing the offspring of any stranger; if he had unholy desires and gratified them he told himself these were his own personal affairs; surely there was no higher authority than himself to whom he should give an account. By a subtle line of reasoning he arrived at the conclusion that God had implanted these desires in his nature and that to gratify them would not be sin. But the woman must give an account to him and he would have no such standard for her. Whatever impulses she had she must repress unless such desire was for him alone. And today we are reaping the pernicious results of this teaching. Men complain, and justly sometimes, that their wives are mere automatons who submit to their caresses because they believe it to be their wifely duty. Whose is the fault? Is not man reaping what in selfishness and distrust he has sown?

Woman holds in her own little hand the destiny of the nation; and everywhere that her voice is heard she is declaring that the double standard must go. .It is man-made, not God-given, and is no more to be tolerated than murder. Thou shalt not commit adultery was written on Mount Sinai along with the other commandments that most men believe to be God-given, and if there was any provision made whereby man was granted certain privileges not given to woman it must have remained a secret among men from that day to this, for the Scriptures fail to record it. When humanity was turned from the Garden of Eden with bowed heads man and woman went out together.

The double standard was of man's making; the single standard must be the work of woman and much of this work must begin with herself. Not many years ago there was but one profession to which woman dared aspire, that of wife and mother. She could be either a wife or an "old maid," and so strong was the sentiment for marriage that few women had the courage to carry this term of reproach through life if an opportunity presented itself for her to become a wife. Today she is free to marry or remain single as she may choose. There is now no profession that she may not enter. She has proven in many instances, in the short time since her "partial emancipation," that she is the equal of man. If she finds a man whom she can love and respect she marries; if she does not she becomes independent and unblushingly calls herself a "bachelor-girl."

With this freedom has come to woman the knowledge that there can be no greater crime than giving herself, soul, mind and body, to a man for whom she feels no genuine affection. She has begun to think of the man she is to marry as the father of her children and for their sakes he must not fall below her standard of ideals. She is realizing that a child, to receive the full measure of life, must be born of parents who are "one" in the truest and fullest sense of the word. She is beginning to feel that she may not degrade herself in the marital relation with a man for whom she feels only gratitude and sympathy without imparting in a measure this degradation to the child she would mother. She is awakening to the fact that sex is the basic stone of all civilization and that there is no virtue in trying to crush out of humanity the fundamental principle that God implanted there for the perpetuation of the race.

The progress of a nation is measured by the standard of its ideals and the ideals most lasting are those that we embrace in our early youth. Realizing this the mother will teach her baby boy that he is just as pure as his sister and that the great Judge of all will not excuse him even though the world does. She will feel, and make him feel, that his chastity is the dearest thing in life to her. The home will be made as attractive for the son as for the daughter; she will not permit him to roam the streets alone until his character is formed, any more than she will permit her daughter to do so. With line upon line and precept upon precept, she will teach him unblushingly that passion is God-given, but that he can and must guide the virility of sex into other channels than that of desire, until such time as he may be able to live holily with his life mate.

Man's share in eradicating the double standard is very important since his work can begin today. There are a few good men who have led pure lives—statistics show that one in every ten men has not contracted any social disease between the age of one and thirty-five. The physicians of character will teach and preach that continence on the part of man is not impossible.

If he is clean minded he will refuse to listen to smutty jokes or laugh at the escapades of licentious men. The man of today must remember that under existing conditions his bride sees life from an exactly opposite angle from which he views it. He has gloried in what he pleased to term his "manhood;" she has sought to suppress everything in her life that related to sex. He knows all the mysteries of life; she knows nothing. He has won her heart and hand; now he must win her body. In winning her body he will have to exercise tenderness and patience. He may not change her views in a week or a month, and if he disregards them it is only a matter of time when she will come to despise him as an unfeeling brute who is incapable of anything but animal lust. If only he would realize that in this regard she is an unsophisticated child and that as such he must teach and wait patiently for her development, there would be fewer sad-eyed women.

### WHAT SHALL THE NEW STANDARD BE?

It is quite safe to say that the new standard will be as unlike the old as was the law of Moses, which claimed an eye for an eye and a tooth for a tooth, from that of the Christ which enjoined the love of one's enemies. It will not tolerate for a moment a loose standard applied to men nor will it accept the merciless standard that applies to the unfortunate woman of today, for that fails to take notice of those who deliberately choose a life of sin and those who are betrayed. Again the present standard fails to distinguish between those who are satisfied to stay down and those who desire to reform. Forgiveness and reformation are offered for every sinner in the land, but the fallen woman of today finds that unlike Christ the world is much more willing to forgive the Scribes, the Pharisees, the hypocrites than it is to say to her, "Go, and sin no more."

Thanks to the teachings of the Master, the new standard will show mercy to both men and women who wish to turn from their immoral lives.

The rapidly growing idea of a marriage health certificate will require that a man shall go to his bride a healthy man at least. Fathers and mothers properly educated to the terrible results of the double standard will be much more anxious that the man who is to marry the daughter of their hearts be sound physically than that he be a moral reprobate with a fat bank account.

Society will cease to tolerate a law that permits a man to betray and befoul the life of some innocent, trusting girl and then refuse to share her shame. He will be regarded as a brute far more dangerous than a mad dog turned loose in the community and every home will be closed against him just as it is closed today to the woman whom he betrays.

